# Molecular Imprinting of Macromolecules for Sensor Applications

**DOI:** 10.3390/s17040898

**Published:** 2017-04-19

**Authors:** Yeşeren Saylan, Fatma Yilmaz, Erdoğan Özgür, Ali Derazshamshir, Handan Yavuz, Adil Denizli

**Affiliations:** 1 Department of Chemistry, Division of Biochemistry, Hacettepe University, 06800 Ankara, Turkey; yeseren@hacettepe.edu.tr (Y.S.); erdoganozgur@hacettepe.edu.tr (E.Ö.); tural@hacettepe.edu.tr (A.D.); handany@hacettepe.edu.tr (H.Y.); 2 Department of Chemistry Technology, Abant Izzet Baysal University, 14900 Bolu, Turkey; fyilmaz71@gmail.com

**Keywords:** macromolecule, molecular imprinting, sensor

## Abstract

Molecular recognition has an important role in numerous living systems. One of the most important molecular recognition methods is molecular imprinting, which allows host compounds to recognize and detect several molecules rapidly, sensitively and selectively. Compared to natural systems, molecular imprinting methods have some important features such as low cost, robustness, high recognition ability and long term durability which allows molecularly imprinted polymers to be used in various biotechnological applications, such as chromatography, drug delivery, nanotechnology, and sensor technology. Sensors are important tools because of their ability to figure out a potentially large number of analytical difficulties in various areas with different macromolecular targets. Proteins, enzymes, nucleic acids, antibodies, viruses and cells are defined as macromolecules that have wide range of functions are very important. Thus, macromolecules detection has gained great attention in concerning the improvement in most of the studies. The applications of macromolecule imprinted sensors will have a spacious exploration according to the low cost, high specificity and stability. In this review, macromolecules for molecularly imprinted sensor applications are structured according to the definition of molecular imprinting methods, developments in macromolecular imprinting methods, macromolecular imprinted sensors, and conclusions and future perspectives. This chapter follows the latter strategies and focuses on the applications of macromolecular imprinted sensors. This allows discussion on how sensor strategy is brought to solve the macromolecules imprinting.

## 1. Molecular Imprinting Methods

**Figure 1:**
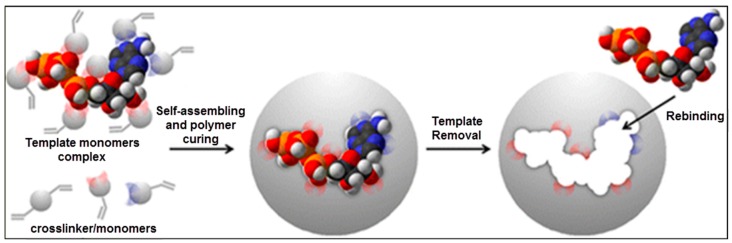
A schematic representation of the molecular imprinting method [14].

One of the first reports about molecular imprinting was published by Wulff and Sarhan in 1972 [1]. Molecular imprinting is one of the most popular methods to present molecular recognition sites and has attracted growing attempts for the preparation of complementary parts of the target molecules [2]. This method mainly relies on the molecular identification reaction that occurs at the surrounding of the target molecule called as a template. As seen in Figure 1, the pre-complex was first formed by the template and functional monomers. The polymerization was completed after the cross-linker and pre-complex interactions that keep the position of the functional groups to bind the template able to produce the molecular recognition sites. At the end of the polymerization stage, the polymeric matrix has specific recognition sites after removing template with suitable desorption agents, so molecularly imprinted polymers (MIPs) are able to bind the template with high selectivity as compared to other competing molecules [3]. MIPs can be produced by several combinations of cross-linkers, functional monomers and solvents [4,5,6]. The quality of the MIPs and their binding features are changed not only via the combination of the mixture, but also the experimental circumstances, such as the initiator type and amount, polymerization temperature, interaction mechanisms, and so on [7,8,9,10,11,12,13].

It is generally supposed that the template acts as a critical molecule and the other compounds (cross-linkers, functional monomers, and solvents) should be selected based on the chemical and physical features of the template [15]. Furthermore, the stability as binding strength of the target molecule of the imprinted polymers is optimized by varying the monomer and cross-linker composition [16].

**Figure 2:**
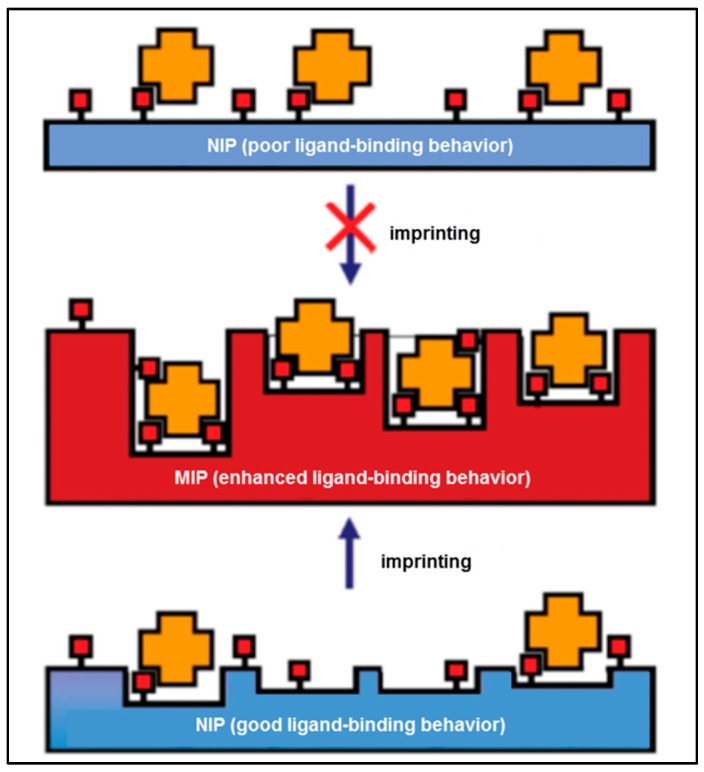
A schematic illustration of the alternative imprinting hypothesis [17].

On the basis of these facts, Baggiani et al. offered an alternative perspective to the molecular imprinting method [17]. As illustrated in Figure 2, the existence of the template in the pre-complex mixture helps to improve interactions that pre-exist in a non-imprinted polymer (NIP). As a result, if the NIP does not bind the target molecule, the MIP will display a weak imprinting effect. On the other hand, if the NIP binds the target, the MIP will display a strong imprinting effect.

They provided a library in the absence of any template to verify their hypothesis. This library was screened for various possible ligands, and the composition of the best-binding NIP yielded the MIP with outstanding binding features. The scanning of the numerous polymers authorized a definite correlation between the binding features of the NIP and MIP libraries [17]. MIPs have been synthesized successfully using different types of molecular imprinting methods including surface imprinting, micro-contact imprinting and epitope imprinting and these polymers have been used in many applications such as purification [18], isolation [19], chiral separation [20], catalysis [21] and in sensors [22]. 

## 2. Developments in Macromolecular Imprinting Methods

Biological molecules such as amino acids, nucleic acids etc. are macromolecules which display significant biological activities and they can be classified as organic molecules [23,24]. The structures of macromolecules are often uninformative about function. Owing to this wide range of functions, detection of proteins, enzymes, nucleic acids, and cells has attracted a huge interest in the high-speed development of biomedicine and proteomics, and other studies as well [25]. 

Even though the use of molecular imprinting method for the detection of macromolecules has a number of advantages, however it also has some drawbacks as well. Firstly, desorption and recognition of the template is the earliest point in the macromolecular imprinting method because rebinding capacity of the extremely cross-linked forms of the MIPs with asymmetric configuration is reduced. Then, the template cannot be removed smoothly from the imprinted cavities. Thus, a decrease of the adsorption and desorption processes occurs. Desorption efficiency of the template in the case of imprinting of macromolecules is low due to the large size of the macromolecule. Nanomaterials can be used to overcome these problems, because nanomaterials have high surface area and volume ratio and also most of the interacting sites are exposed on the surface. Thus, after easy removal of the template, higher rates of adsorption can be achieved [26,27,28]. Different types of imprinting methods also help to figure out any kind of problems in the imprinting of macromolecules, which are summarized below. 

### 2.1. Surface Imprinting Methods 

Surface imprinting methods help reduce the mass transfer resistance in macromolecule imprinting processes [29]. In recent years, surface imprinting, as a significant progress in macromolecular imprinting methods, has gained great attention, especially in separation, sensor and diagnostic applications [30]. 

These methods can be divided into two sub-classes—top-down and bottom-up—based on the location where the polymerization occurs. In a top-down process, the template is bound to a support, which is removed after formation and the interaction areas on the polymer surface retreat [31,32]. There are some studies that show the production of surface imprinted nanomaterials [33,34,35]. The support utilize to immobilize the template enhances the substrate on which the polymer is inserted and improves the substrate in the bottom-up process. In this method, the removal of the template has to be accomplished impressively to obtain imprinted materials. The bottom-up process has been employed for the preparation of macromolecule imprinted microparticles [36,37,38], quantum dots [39], magnetic nanoparticles [40,41], carbon nanotubes [42], and gold electrodes [43,44]. A comparable method has been employed to produce sites imprinted with macromolecules [45,46]. 

**Figure 3:**
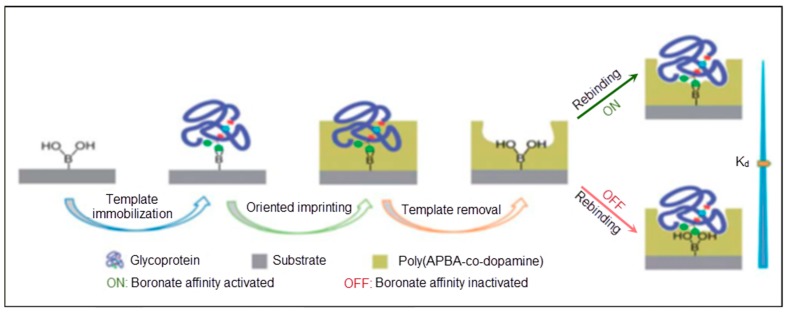
A schematic illustration of the surface imprinting of glycoproteins [47].

Recently, Wang et al. [47] offered a surface imprinted model to detect glycoproteins, as illustrated in Figure 3. Dopamine and *m*-aminophenyl boronic acid were deposited on the sensor surface. After template removal, the imprinted cavities demonstrated recognition ability with high affinity in basic medium. The dissociation constant was calculated as 6.6 × 10^–9^ at pH 9.0 and 2.7 × 10^–7^ at pH 3.0 for horseradish peroxidase (HRP). 

Further examples are the ionic liquid-modified graphene-based sensor [48], quartz crystal microbalance (QCM)-based sensor [49] and molecularly imprinted micro-particles for macromolecule detection [50]. 

### 2.2. Micro-Contact Imprinting Method 

Another solution for the problem of imprinting of large, labile and non-rigid macromolecules has been figured out by researchers using a micro-contact imprinting method. The micro-contact imprinting method needs a small amount of template mass, which is efficiently used as a monolayer. Some scientists have applied a stamp to place the template moiety during the imprinting process [51,52,53]. They fabricated a single layer of the template with the functional monomer on a glass slide and then attached this layer onto a glass slide that has the cross-linker. At the end of the UV polymerization, the glass slide was smoothly removed from the surface of the polymer, which demonstrated the desired recognition features. 

The operative site of the template that helps to produce interactions with molecules in the polymer was decreased in the imprinted polymeric films [54,55]. Silicon wafers, glass slides, gold surfaces were employed as a stamp to fabricate imprinted polymeric matrices. Micro-contact imprints of some templates such as creatine kinase, lysozyme, RNase A, myoglobin and C-reactive protein were formed by micro-contact imprinting methods [56,57,58,59,60]. 

### 2.3. Epitope Imprinting Methods 

Epitope imprinting methods have become a new method for the identification and separation of target molecules. According to this approach, a region of a macromolecule is employed as a template instead of the whole macromolecule during the imprinting process. Thus, small peptide sequences could recognize a whole protein [61,62,63,64] or F_ab_ fragments were used to detect human immunoglobulin G as a model of the protein fragment [65]. Epitope imprinting methods have several advantages such as organic solvents can be used, and the costs of the peptides are lower than proteins and they can be produced in very pure form, so increased selectivity can be achieved [66]. 

**Figure 4:**
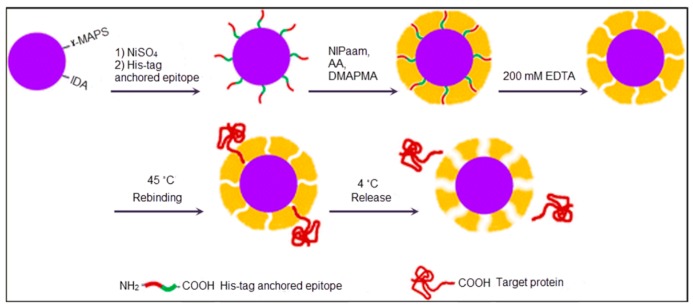
A schematic representation of the epitope imprinting method [76].

Thermodynamic considerations imply that the non-rigid template usage leads to less clear recognition sites in MIPs [67], so the composition includes the macromolecule in the polymerization by framing and also they have some disadvantages such as low specificity and insufficient reproducibility [68,69,70,71]. Mosbach et al. also proved the efficient recognition of short oligopeptides by MIPs [72,73,74]. In order to resolve the recognition problem, Rachkov and Minoura suggested imprinting only fixed limited areas of recognition sites [75]. Epitope imprinting and a temperature- dependent capture and release process were performed by Li and coworkers [76]. In their study, SiO_2_ nanoparticles were immobilized by glycidoxypropyltrimethoxysilane-iminodiacetic acid and 3-(trimethoxysilyl)propyl methacrylate to promote the template modification, as reflected in Figure 4. After immobilization of a His-tag-anchored epitope of human serum albumin, polymerization was conducted using N-isopropylacrylamide as a monomer to obtain a thermosensitive imprinted shell. Finally, the template was removed by ethylenediaminetetraacetic acid, and the formed epitope imprinted sites could capture template at 45 °C and release it at 4 °C. 

## 3. Macromolecular Imprinted Sensors

Sensors are tools for the analysis of molecules to obtain their composition, structure and function by converting biological responses into electrical signals [77]. They should fundamentally comprise a transducer (electrochemical [78], piezoelectric [79], or optical [80]) and a recognition molecule, which interacts with an analyte. A number of optical sensing studies have been performed on sensors involving chemiluminescence [81], fluorescence [82], light absorption [83], and reflectance [84] that can be classified as label-based and label-free. While label-based sensing is highly sensitive, and the limit of detection is also very low, it is limited to hard labelling processes that may also hinder the molecule function [85]. On the contrary, label-free sensing reacts in the natural forms and depends on the measurement of refractive index changes. As a result, label-free sensing is comparatively simple and cheap, and also permits one to perform quantitative and kinetic analyses for molecular recognition [86]. The recognition molecules are significant components of sensors because they are responsible for the capture of the target molecules, so the recognition molecule selection is completely based on the target molecule. The recognition molecules are selected according to their high affinity and also stability to the target molecule. Chemical sensors can be sorted according to the recognition molecules used. The advantages and disadvantages of some recognition molecules in sensors are as listed in Table 1 [87].

### 3.1. Enzyme Imprinted Sensors 

Molecular imprinting processes lock the enzyme into a certain conformation that is favorable for catalysis. In this rigid form, enzymes remain more active and selective in the presence of organic solvents than they are in aqueous media. This makes a large number of applications in chemical, pharmaceutical and polymer industries possible [88]. 

**Figure 5:**
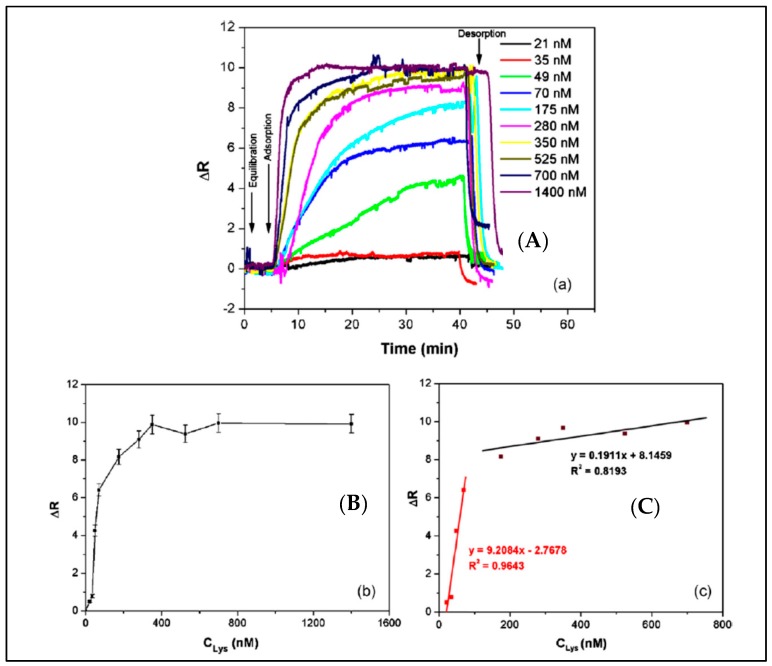
The detection of lysozyme with lysozyme imprinted SPR sensor: (**A**) concentration dependence of lysozyme imprinted SPR sensor, (**B**) concentration versus SPR sensor response, (**C**) linear regions [89].

A surface plasmon resonance (SPR) sensor with lysozyme-imprinted nanoparticles was designed by Sener et al. as a recognition element to detect lysozyme [89]. They immobilized lysozyme imprinted nanoparticles onto the SPR sensor surface. As shown in Figure 5, this SPR sensor could perform in both aqueous and natural solutions. The concentration of lysozyme was as low as 32.2 nM. They also calculated that the limit of detection (LOD), association (K_a_) and dissociation (K_d_) values as 84 pM, 108.71 nM^−1^ and 9.20 pM, respectively.

**Figure 6:**
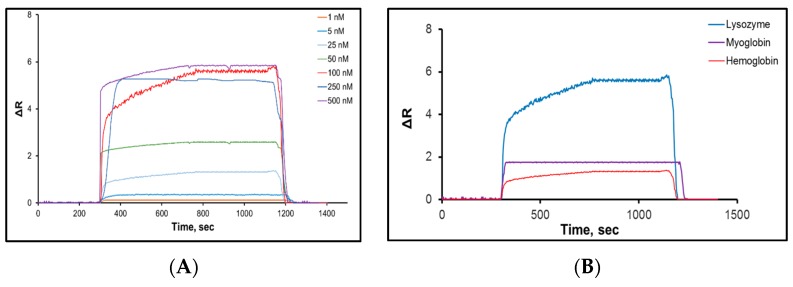
The (**A**) concentration dependency and (**B**) selectivity experiments of SPR sensor [90].

Saylan and colleagues developed an SPR-based sensor to detect lysozyme with hydrophobic poly(N-methacryloyl-L-phenylalanine) nanoparticles [90]. Various concentrations of lysozyme solutions were used to calculate kinetic and affinity coefficients (Figure 6A). The equilibrium and adsorption isotherm models of interactions between the lysozyme solutions and the SPR sensor were determined and the maximum reflection, association and dissociation constants were calculated by a Langmuir model as 4.87 nM, 0.019 nM and 54 nM, respectively. Selectivity studies of the SPR sensor were performed with competitive agents like hemoglobin and myoglobin (Figure 6B). The results showed that the SPR sensor could detect lysozyme in lysozyme solutions with high accuracy, good sensitivity, in real-time, label-free, and with a low LOD value of 0.66 nM.

**Figure 7:**
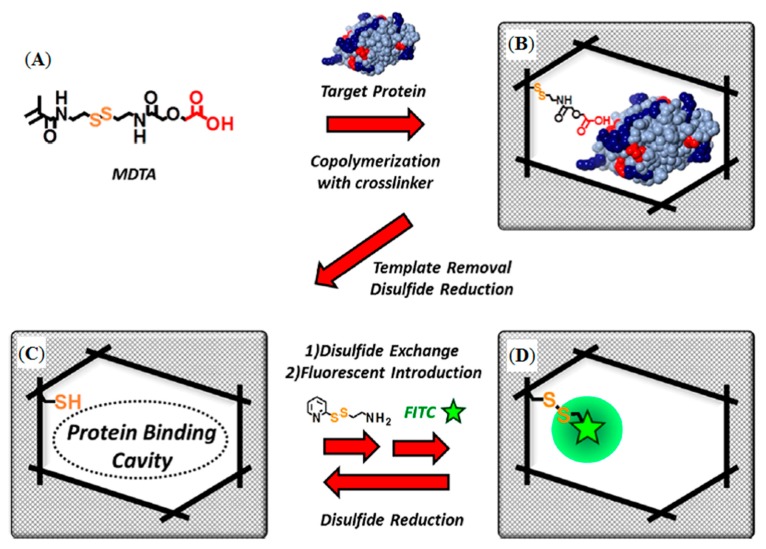
Schematic illustration of the preparation of lysozyme-imprinted polymer: (**A**) ({[2-(2-methacrylamido)ethyldithio]ethylcarbamoyl}methoxy)acetic acid structure, (**B**) protein-imprinted polymer preparation, (**C**) binding cavity created by the disulfide linkage reduction and (**D**) fluorophore introduction by the disulfide linkage reformation [91].

Sunayama et al. reported a new functional monomer which could convert the macromolecule identification signal event into a fluorescent signal [91]. They prepared lysozyme-imprinted polymers which were organized on glass substrates by copolymerization of a functional monomer, and cross-linker, in the presence of lysozyme (Figure 7A,B).

In the first post-imprinting modifications after the removal of lysoyzme resulted in the creation of the lysozyme-binding cavities, the residual (ethylcarbamoylmethoxy)acetic acid moiety within the cavities was removed by reduction (Figure 7C). In the second post-imprinting modification, the disulfide linkage was reformed using aminoethylpyridyl disulfide to introduce aminoethyl groups (Figure 7D), followed by treatment with fluorescein isothiocyanate to label the amino groups within the cavities, in the third post-imprinting modification. The reusability and tunability of the prepared MIPs were evaluated by fluorescence measurements to demonstrate the effectiveness of the proposed method. All studies about enzyme detection were summarized in Table 2 according to the different parameters.

### 3.2. Antibody/Antigen Imprinted Sensors

Molecular imprinting methods have become a straightforward and versatile way of making synthetic receptors that can recognize target molecule with affinity and selectivity. This has led to them being called antibody mimics, and they can be used in sensor systems to detect antigens and also antibody molecules with higher physical and chemical stability than their biomolecule counterparts, which are restricted in stability under external conditions besides being expensive. Some studies in which clinically significant antigen/antibody molecules were selected as target molecules are summarized below. 

A cyclic citrullinated peptide antibody-imprinted SPR sensor was prepared by Dibekkaya and collaborators to detect cyclic citrullinated peptide antibodies [92]. They used different concentrations of cyclic citrullinated peptide antibodies for real-time detection. They also calculated LOD, K_a_ and K_d_ constants of 0.177, 0.589 RU/mL and 1.697 mL/RU, respectively. According to their results, the cyclic citrullinated peptide antibody-imprinted SPR sensor can be employed several times for cyclic citrullinated peptide antibodies detection with remarkable selectivity and sensitivity.

**Figure 8:**
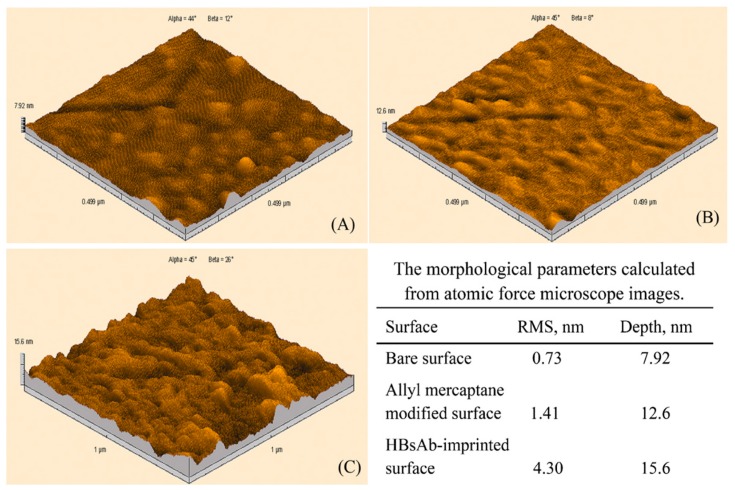
Atomic force microscopy pictures of (**A**) non-modified, (**B**) allyl mercaptan-modified, (**C**) hepatitis B surface antibody-imprinted SPR sensor [93].

**Figure 9:**
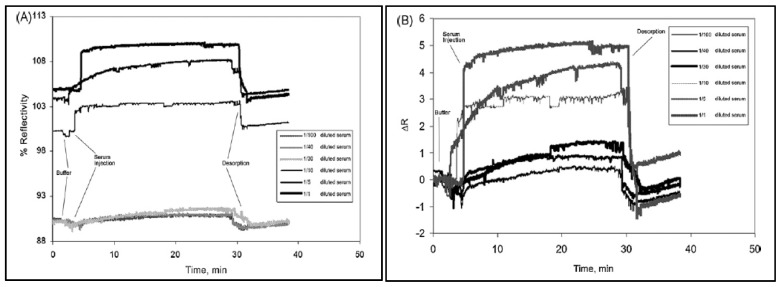
Sensorgrams for the interaction between hepatitis B surface antibody positive human serum and hepatitis B surface antibody imprinted SPR sensor (**A**) reflectivity and (**B**) ΔR vs. time. [93].

Uzun et al. developed a hepatitis B surface antibody imprinted film on a SPR sensor and measured hepatitis B surface antibody concentrations in human serum [93]. They first characterized the SPR sensor by atomic force microscopy (Figure 8) and then performed kinetic studies with different concentrations of hepatitis B surface antibody positive human serum samples (Figure 9). The LOD value was found to be 208.2 mIU/mL. 

**Figure 10:**
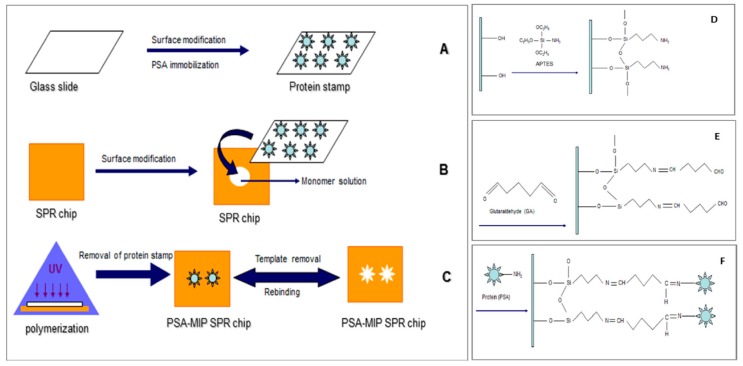
(**A**) Glass slides preparation, (**B**) surface modification of SPR sensor, (**C**) micro-contact imprinting of prostate specific antigen, (**D**) surface modification of glass slides, (**E**) amino groups activation on glass slides and (**F**) prostate specific antigen immobilization onto the glass slides [94].

A micro-contact imprinting-based SPR sensor to detect prostate specific antigen (PSA) was developed by Ertürk et al. [94]. As shown in Figure 10A–F, they prepared the SPR sensor by UV polymerization based on the micro-contact imprinting method. They detected PSA in a 0.1–50 ng/mL concentration range with a LOD value of 91 pg/mL. They also analysed 10 clinical samples using their PSA-imprinted SPR sensor and indicated around 98% accuracy between their results and those obtained by a commercial ELISA method.

**Figure 11:**
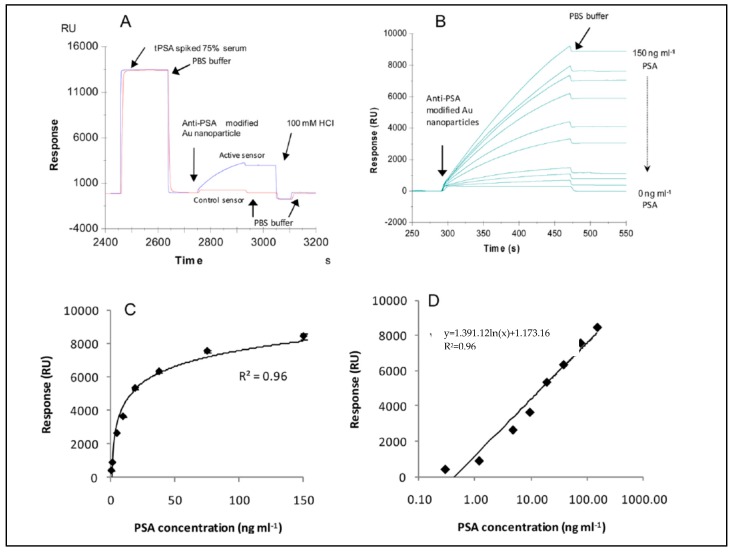
The (**A**) detection of total PSA antibody-modified gold nanoparticles after the PSA injection, (**B**) 4.69, 1.17, 0.29, 0 ng/mL on total PSA antibody immobilized surface and 150 ng/mL on IgG-immobilized surface, (**C**, linear, **D**, log scales) the calibration curves that came by from the assay [95].

Uludag et al. described a system to detect total prostate-specific antigen in human serum samples with SPR and QCM sensors [95]. They performed a sandwich assay using antibody- based nanoparticles and detected 2.3 ng/mL and 0.29 ng/mL total prostate-specific antigen concentrations in human serum (Figure 11). They also showed that their results correlated well with those of a QCM sensor.

**Figure 12:**
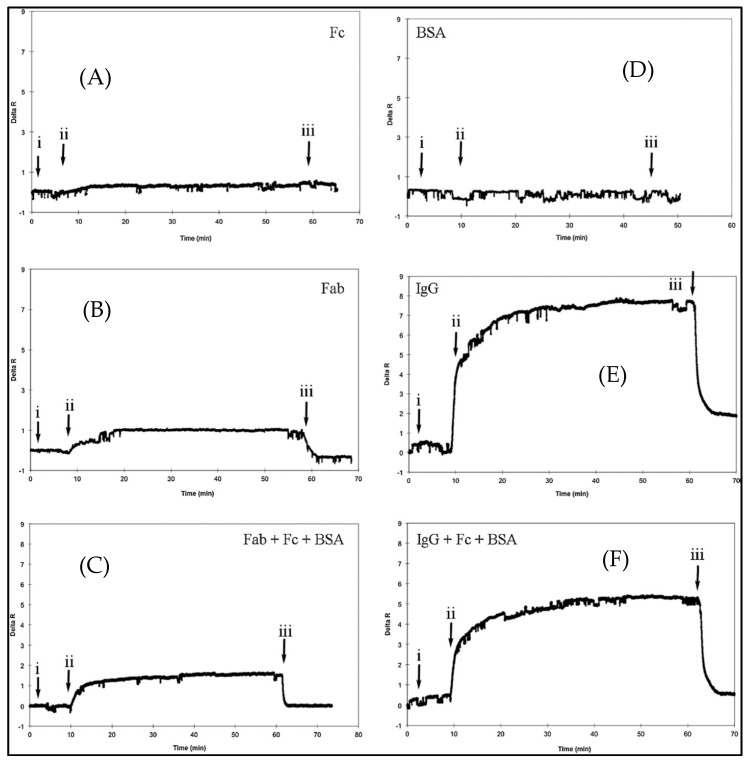
The selectivity SPR sensor (**A**–**F**): (i) Equilibrium by phosphate buffer, (ii) the analyte solutions application, and (iii) desorption with phosphate buffer that has 1 M NaCl [65].

Ertürk and coworkers prepared a F_ab_ fragments-imprinted SPR sensor to detect human immunoglobulin G (IgG) [65]. They digested IgG molecules with papain and concentrated them by fast protein liquid chromatography. They formed a complex between F_ab_ fragments and the specific monomer, and then prepared nanofilms on the SPR sensor surface using a cross-linker and functional monomer. The group carried out IgG detection studies using different concentrations of aqueous IgG solutions. They also performed experiments to verify the selectivity of the F_ab_-imprinted SPR sensor by using bovine serum albumin, IgG, F_ab_ and F_c_ fragments. The SPR sensor has no response to bovine serum albumin (Figure 12D) and F_c_ (Figure 12A) solutions, while has specific responses to F_ab_ fragments (Figure 12B) and IgG (Figure 12E) with higher affinity. They also performed with pre-mixed protein solutions that contained F_ab_/F_c_/BSA (Figure 12C) and IgG/F_c_/BSA (Figure 12F) for a second confirmation of SPR sensor selectivity.

**Figure 13:**
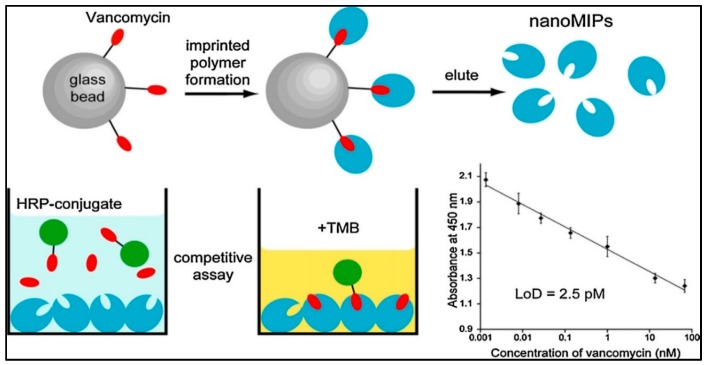
The schematic model of solid phase imprinting to produce artificial antibodies [96].

Chianella et al. modified the common ELISA test by replacing the antibodies with molecularly imprinted nanoparticles as depicted in Figure 13 [96]. They achieved detection of vancomycin in studies on competition with a horseradish peroxidase−vancomycin conjugate. Their study was able to detect vancomycin in aqueous and blood plasma solutions in the range of 0.001−70 nM with LOD value of 0.0025 nM. They showed that the sensitivity of the study was three times higher than the ELISA predicated on antibodies. 

As indicated by Türkoğlu et al. polymeric nanoparticles were fixed to a SPR sensor to detect human IgG in human serum [97]. They performed detection studies by utilizing aqueous IgG solutions at different concentrations. They found a K_a_ value as 1.810 µg/mL with a high correlation coefficient (R^2^ = 0.9274). They also showed that the best adsorption model showing the interaction between the SPR sensor and IgG was the Langmuir adsorption isotherm. All studies about antibody and antigen detection were summarized in Table 3 according to the different parameters.

### 3.3. Protein Imprinted Sensors

Molecular imprinting techniques are proven to work well with small molecules but they are challenging for larger molecules like proteins because of their complexity, conformational flexibility and solubility. However it has been shown that it is possible to prepare protein-imprinted films or nanoparticles for various applications including sensors, by i.e. surface imprinting, epitope imprinting, metal-ion coordination, or using natural nontoxic and biocompatible polymers as facile and green approaches [98]. 

**Figure 14:**
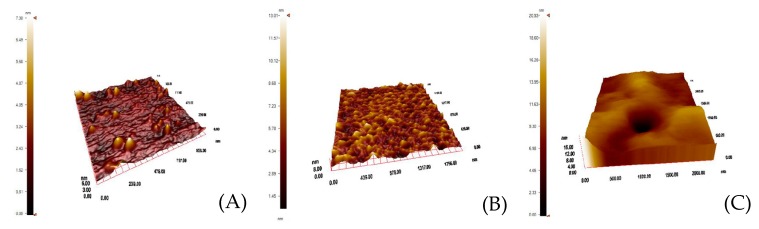
The characterization of the glass and SPR sensor: The atomic force microscopy images of (**A**) myoglobin immobilized glass, (**B**) bare SPR sensor, (**C**) myoglobin imprinted SPR sensor [60].

Osman et al. developed a SPR sensor combined with molecularly imprinted synthetic receptors. A myoglobin-imprinted polymeric film was integrated onto the SPR sensor and characterized by atomic force microscopy and contact angle measurements (Figure 14). Then they evaluated the detection behaviors of the developed sensor for myoglobin with myoglobin solutions in different concentrations scale in phosphate buffer and serum. The LOD value of the SPR sensor was calculated as 26.3 ng/mL [60].

Moreira et al. described a novel use of the polymeric film poly(*o*-aminophenol) that was made responsive to a specific protein [99]. This was accomplished through electropolymerization of aminophenol with protein. Proteins embedded in the outer surface of the polymeric film were digested by proteinase K and then washed out thereby creating empty sites. The films acted as biomimetic artificial antibodies and were produced on a gold screen printed electrode, as a step towards disposable sensors. The sensors displayed linear responses to myoglobin down to 4.0 and 3.5 g/mL with LOD values of 1.5 and 0.8 g/mL. 

**Figure 15:**
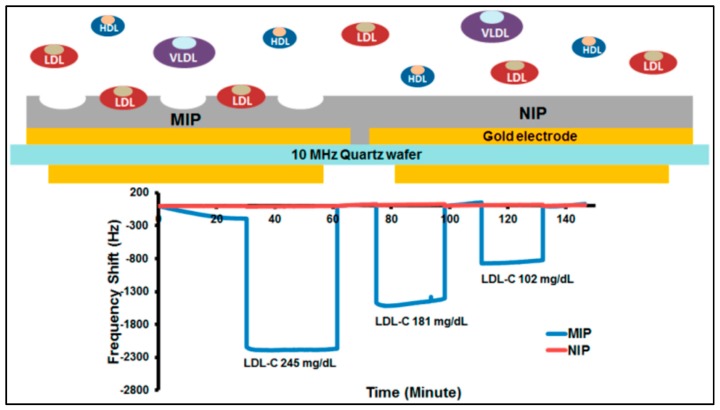
The scheme and recovery rates of the QCM sensor [101].

Chunta and co-workers synthesized MIPs for the detection of low-density lipoprotein [100]. They examined that the ratios of monomers acrylic acid, methacrylic acid, and N-vinylpyrrolidone and analyzed by samples by QCM (Figure 15). The QCM sensor had an accuracy of 95−96% at the 95% confidence interval with 6−15% precision. Their results showed that QCM sensor responses were in agreement with those of standard methods. 

Reddy et al. studied hydrophilic molecularly imprinted hydrogels to detect bovine hemoglobin and insulin [101]. They investigated the specific binding capacity of four different functional monomers. After finding the optimal conditions, they synthesized the hydrogel from acrylamide functional monomer that was found to have the best specific adsorption capacity.

**Figure 16:**
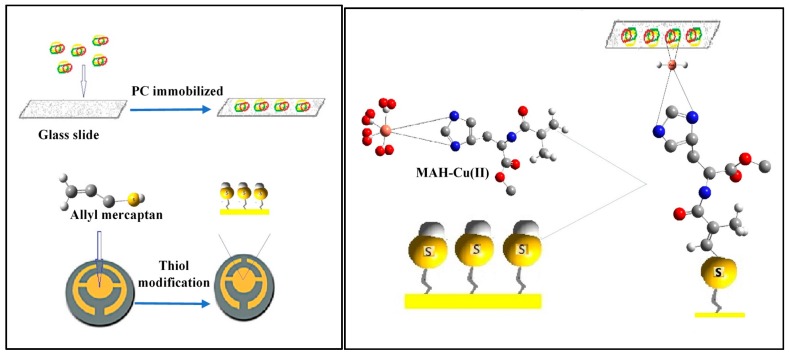
Schematic representation of the protein C-imprinted QCM sensor [102].

Bakhshpour and coworkers detected protein C in human serum by QCM [102]. The protein C micro-contact imprinted polymeric film was prepared on a glass surface. Then, the QCM sensor was prepared using suitable functional monomers and cross-linker with copper (II) ions. The polymerization was performed under UV light for 20–25 min (Figure 16). Detection of protein C was studied in a concentration range of 0.1–30 μg/mL. The LOD value for protein C analysis was determined as 0.01 µg/mL. 

Another study was published by Wang et al. was based on the use of a carbon electrode to fabricate an imprinted electrochemical sensor for bovine hemoglobin detection [103]. They examined the fabrication parameters such as the concentration of pyrrole, scan cycles and rates of the imprinted electrochemical sensor. After optimization, they showed that the imprinted electrochemical sensor had fast rebinding features that helped detect bovine hemoglobin with a high correlation coefficient (R = 0.998) and low LOD value (3.09 × 10^−11^ g/L). They also revealed that the imprinted electrochemical sensor had a very good selectivity and stability, and can be used for immunoassays and clinical applications.

Wang and collaborators also constructed a potentiometric myoglobin or hemoglobin sensor using self-assembled monolayers [104]. They produced a sensing surface on the sensor by using self-assembled monolayers and template molecules. Their results showed that the sensor could detect myoglobin or hemoglobin proteins in the presence/absence of other proteins in aqueous solution. 

Moreira and co-workers built a self-assembled monolayer sensor for myoglobin detection [105]. They found the optimum conditions in HEPES buffer. The LOD value was found to be 1.3 × 10^−6^ mol/L. They also showed the imprinting effect by non-imprinted particles with no ability to detect myoglobin. The sensor had a good selectivity towards other molecules such as creatinine, sacarose, fructose, galactose, sodium glutamate, and alanine. 

Çiçek et al. prepared bilirubin-imprinted polymeric films on a QCM sensor [106]. They prepared sample solutions in the concentration range of 1–50 g/mL to determine the relationship between the bilirubin sample solutions and the QCM sensor response. They also calculated the LOD and LOQ values as 0.45 and 0.9 g/mL, respectively.

Dechtrirat and collaborators prepared a MIP film on a gold-based transducer surface for cytochrome C detection in aqueous solution [107]. They used a combination of the epitope and surface imprinting approaches. They characterized the recognition capabilities of the films and confirmed that the MIP film was able to detect cytochrome C selectively. They also showed that the MIP film could discriminate even single amino acid mismatched sequences on the target peptide. All studies about protein detection were summarized in Table 4 according to the different parameters.

### 3.4. Nucleic Acid Imprinted Sensors 

Nucleic acids are suitable molecular recognition elements for the detection of DNA and RNA molecules owing to their potential to form Watson-Crick pairs. Beyond this, the use of nucleic acids as biorecognition elements was extended to aptamers, which bind non-nucleotide molecules as well with a broad scope. The nucleic acid or aptamer-imprinted systems offer several advantages in terms of improved selectivity with a protection against enzymatic and chemical degradation [108]. 

**Figure 17:**
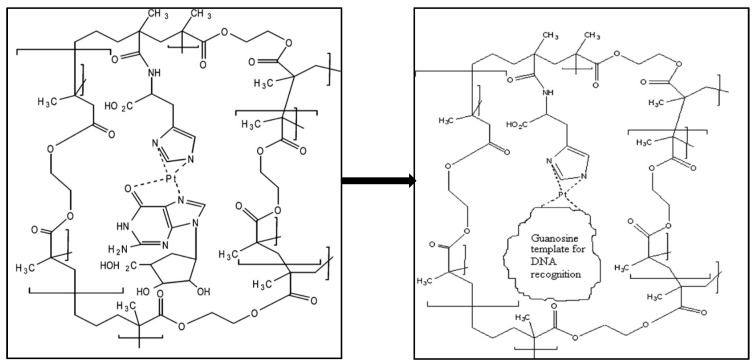
Scheme of the formation of guanosine-imprints on SPR sensor surface [109].

Diltemiz et al. developed a SPR sensor by using methacrylamidohistidine-platinum (II) for recognition of DNA [109]. They reported that not only the detection of guanosine guanine and but also assay DNA sequencing is possible with the MIP SPR sensor. The formation of guanosine-imprints on SPR sensor surface is shown in Figure 17. Briefly, they cleaned the SPR sensor surface and dipped it into monomer solution for 24 h. After that, immobilized surface was dipped in the mixture that included the metal-chelate monomer, crosslinker and initiator for the polymerization. They carried out the polymerization at room temperature by using UV light for 4 h.

Ersöz and colleagues prepared an imprinted QCM sensor using methacryloylaminoantipyrine-Fe(III) as a metal-chelating monomer to detect 8-hydroxy-2′-deoxyguanosine [110]. They used a photograft surface polymerization technique to synthesize the imprinted film by UV light. They determined the K_a_ value of the imprinted QCM sensor as 78,760 M^−1^. They also analysed 8-hydroxy-2′-deoxyguanosine levels in the blood serum of a breast cancer patient with the imprinted QCM sensor and found a 8-hydroxy-2′-deoxyguanosine level of 0.297 µM could be detected in 20 min.

Diltemiz and her research group also developed QCM sensors to determine thymine by using methacryloylamidoadenine monomer [111]. They investigated the binding affinity of the QCM sensor by using the Langmuir isotherm and calculated K_a_ value as 1.0 × 10^5^ M^−1^. Their results showed that the QCM sensor had homogeneous binding sites for thymine. They also reported the selectivity of the QCM sensor according to single-stranded DNA, uracil and single-stranded RNA binding experiments. They obtained the initial slope for single-stranded DNA, uracil and single-stranded RNA as −9400 Hz mmol/L, −4700 Hz mmol/L and −2200 Hz mmol/L, while the initial slope of the curve was −11,800 Hz mmol/L.

Taghdisi et al. developed an electrochemical sensor that depended on the form of a dual-aptamer-complementary strand of aptamer conjugate, gold electrode and exonuclease I to detect myoglobin [112]. They observed a weak electrochemical signal in the absence of myoglobin due to the intact form. After the addition of myoglobin, they also observed a strong electrochemical signal because the dual-aptamer left the complementary strand of aptamer and bound to myoglobin. The electrochemical sensor displayed the high selectivity for myoglobin with low LOD value (27 pM). 

Li and colleagues also fabricated an electrochemical sensor to detect myoglobin [113]. They built the electrochemical sensor by grafting myoglobin-binding-aptamer onto the surface of a gold nanoparticles/arginine-glycine-aspartic/carboxylatedgraphene/glassy carbon electrode. Their results showed the electrochemical sensor has good reproducibility and stability. Their electrochemical sensor was also used in biochemical assays with satisfactory results. All studies about nucleic acid detection were summarized in Table 5 according to the different parameters.

### 3.5. Cell Imprinted Sensors 

Recognition and isolation of cells have particular importance in clinical, diagnostic, environmental and security applications. Their size and density limit the use of traditional methods in complex sample mixtures. Molecularly imprinted sensors prepared with whole cells or epitopes provide rapid cell detection platforms [114].

**Figure 18:**
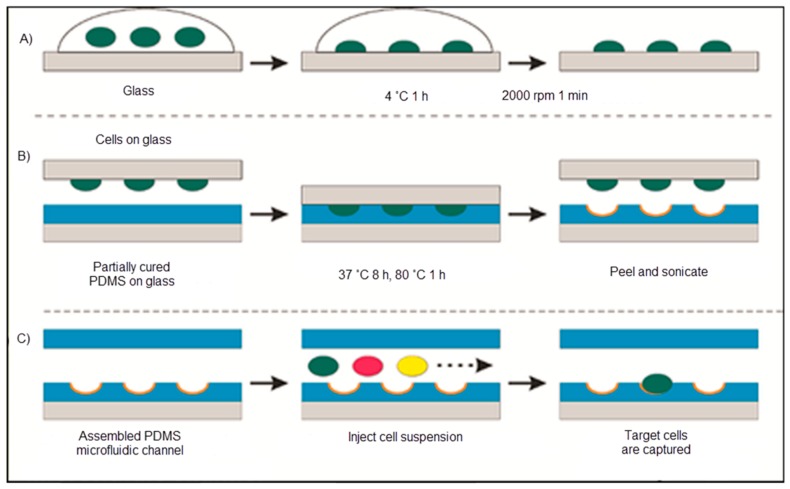
The schematic representation of the cell-imprinted polydimethylsiloxane process: (**A**) The template preparation, (**B**) polymer imprinting with the template and (**C**) cell sorting with the microfluidic chip [115].

Ren and co-workers covered glass with bacteria that was pushed into another glass coated with polydimethylsiloxane. As illustrated in Figure 18, the cell-imprinted polydimethylsiloxane created the resulting surface to favourably capture the imprinted bacteria. They also interpreted this result as powerful proof that chemical interaction acts a superior function in cell sorting with cell-imprinted polydimethylsiloxane films [115]. 

Bers and co-workers synthesized a cell-specific surface-imprinted sensing system that was integrated with a heat transfer-based method to detect cells in cell mixtures [116]. They used a modified hamster ovarian cell line as a model. They also showed that their sensing system distinguished between different types of cells that only differ in the specific membrane protein expression. In addition, they disclosed that the detection sensitivity could be improved via exhibiting the sample surface and removing non-specific cells by purging between consecutive cell exposures.

Eersels et al. used an imprinting method with heat transfer resistance measurements to produce a cell-based sensor for macrophage and cancer cells detection [117]. Their approach was dependent on the difference between the heat transfer resistance at the port of the sensor influenced by the binding of a cell to the imprinted polymeric layer. They noted that the binding of cells caused in measurable extension of heat transfer resistance that indicated the cells acted as a thermally insulating layer. They calculated the LOD value as 10^4^ cells/mL.

Mahmoudi et al. obtained cell-imprinted substrates based on mature and dedifferentiated chondrocytes [118]. They used rabbit adipose-derived mesenchymal stem cells seeded on these cell-imprinted substrates to adopt the specific shape and molecular characteristics of the cell types which had been used as a template for the cell-imprinting. Their data suggested that besides residual cellular fragments, which were presented on the template surface, the imprinted topography of the templates plays a role in the differentiation of the stem cells. All studies about cell detection were summarized in Table 6 according to the different parameters.

### 3.6. Bacteria Imprinted Sensors 

**Figure 19:**
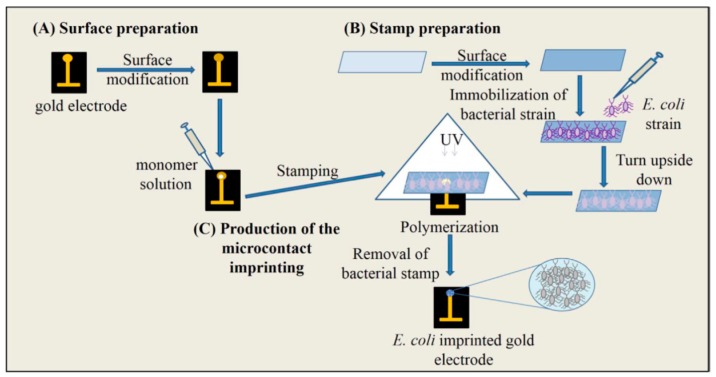
The scheme of the micro-contact imprinting of *E. coli* onto the polymer modified surfaces. The preparation of electrode surface (**A**), bacteria stamps (**B**), production of the micro-contact imprinting (**C**) [120].

There are a number of methods to identify bacteria in different media, however most of them require long incubation times and space for incubators. The need for an efficient, rapid, simple and cheap method for detection of microorganisms is fulfilled by bacteria-imprinted sensors [119]. İdil and collaborators prepared a label-free, selective and sensitive micro-contact-imprinted capacitive sensor for the detection of *Escherichia coli* (*E. coli*) [120]. After preparation of bacterial stamps, micro-contact *E. coli*-imprinted gold electrodes were prepared by using an amino acid-based recognition element, monomers and crosslinker under UV-polymerization (Figure 19). They performed real-time *E. coli* detection within the range of 1.0 × 10^2^–1.0 × 10^7^ CFU/mL and calculated the LOD value as 70 CFU/mL. They also detected *E. coli* with a recovery of 81–97% in samples, e.g. river water.

**Figure 20:**
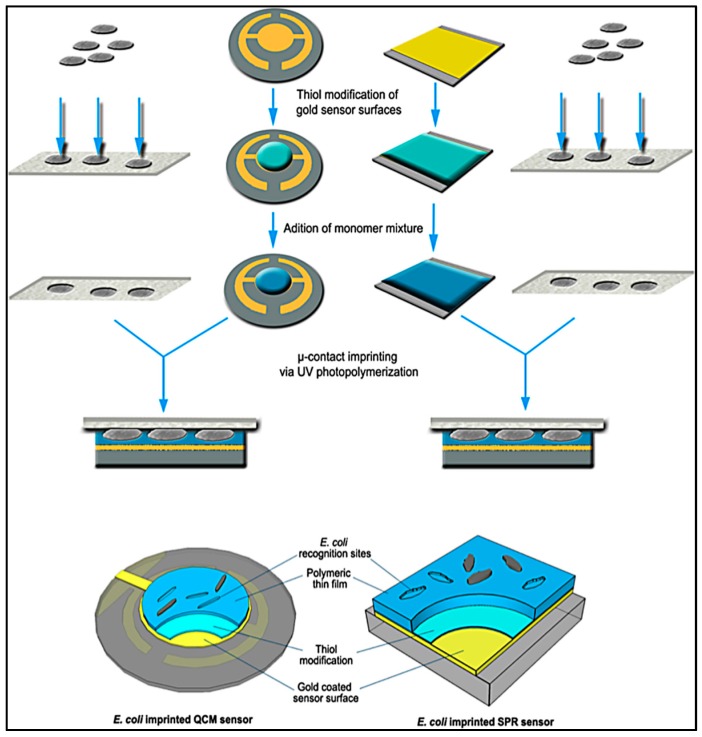
A schematic model of the imprinted SPR and QCM sensors [121].

**Figure 21:**
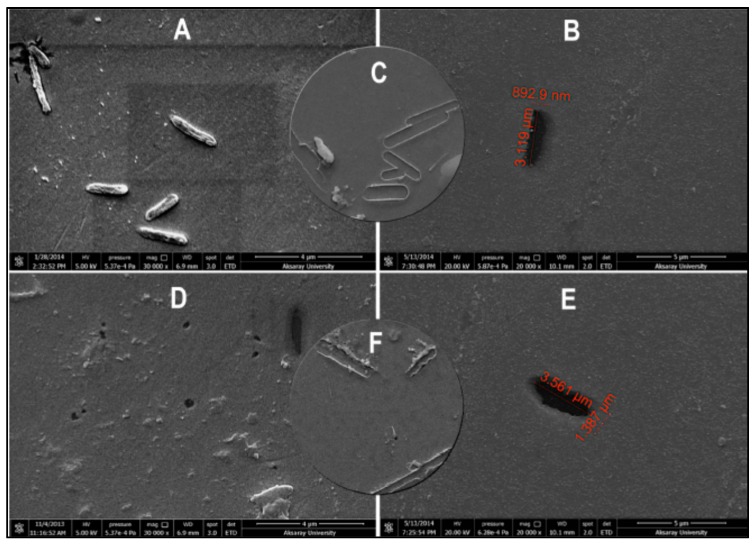
The surface morphologies of *E. coli*-imprinted (**A**–**C**) SPR and (**D**–**F**) QCM sensors [121].

As seen in Figure 20, Yılmaz et al. demonstrated a bacteria detection technique based on a micro-contact imprinting method on both SPR and QCM sensors [121]. N-methacryloyl-L-histidine methyl ester was employed in this study to obtain recognition comparable to that of natural antibodies. They also showed that the characterization of their SPR and QCM sensors by scanning electron microscopy. As seen in Figure 21, scanning electron microscopy images showed that SPR and QCM sensors had different sized imprinted cavities. They claimed that this might give rise to the random orientation of *E. coli* cells during fixation and local immersion distinctions during imprinting because of the rough glass slide surface.

Molecularly imprinted nanoparticles were visualized by Altintas and co-workers for endotoxin detection from *E. coli* with computational modeling [122]. Their process depended on the binding energy between endotoxin and each monomer. They showed that nano-MIPs were produced with functional groups to make the immobilization onto SPR sensor easy. The SPR surface could be regenerated more than 30 times without any significant loss in binding activity which made this method cost effective. 

Hayden and Dickert developed artificial recognition sites for the monitoring of cells with moldable polymers [123]. They choose a mass-sensitive QCM sensor as transducer for the analysis due to its on-line acquisition capability and high sensitivity. The selectivity of the extremely hardy sensor permitted them to separate yeasts, Gram positive and negative bacteria. Their results encouraged them to enlarge the exploration of artificial interaction sites to the micrometer scale.

Wan et al. designed a sensor that contains three quaternized magnetic nanoparticles fluorescent polymer systems to recognize and quantify bacteria [124]. The bacterial cell membranes disrupt the quaternized magnetic nanoparticle fluorescent polymer, generating a unique fluorescence response array. The response intensity of the array was dependent on the level of displacement determined by the relative quaternized magnetic nanoparticles fluorescent polymer binding strength and bacterial cells-magnetic nanoparticles interaction. Their approach has been used to measure bacteria within 20 min with an accuracy of 87.5% for 10^7^ CFU/mL. Combined with UV-VIS measurements, the method can be successfully used to identify and detect eight different pathogen samples with an accuracy of 96.8%.

Qi et al. discussed the preparation of bacteria-based imprinted films on an impedimetric sensor [125]. They choose marine pathogen sulfate-reducing bacteria as a template. They deposited the chitosan doped with reduced graphene sheets, and helped the reduced graphene sheets-chitosan hybrid film as a platform for bacterial fixing. Furthermore, they also deposited a layer of chitosan film to embed the pathogen and then the bacterial template was washed out with acetone. All studies about bacteria detection were summarized in Table 7 according to the different parameters.

### 3.7. Virus Imprinted Sensors 

As viral contamination is a life-threatening issue, rapid and reliable detection of viruses as food borne pathogens, in the pharmaceutical industry and biological warfare conditions has crucial importance. 

**Figure 22:**
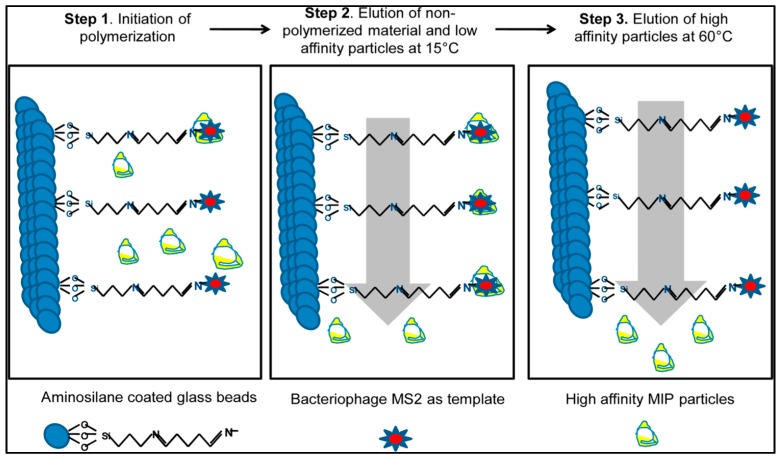
The fundamental of the synthesis by using a solid-phase method [126].

Altintas et al. presented a SPR-based microfluidics system to detect biological factors from water sources [126]. They used a new synthesis method (Figure 22) that was developed by the Cranfield University Biotechnology Group to obtain imprinted nanoparticles based on the existence of bacteriophage MS2 immobilized beads. They separated low and high affinity nanoparticles by using different temperatures and indicated the differences between high and low temperatures [127,128].

**Figure 23:**
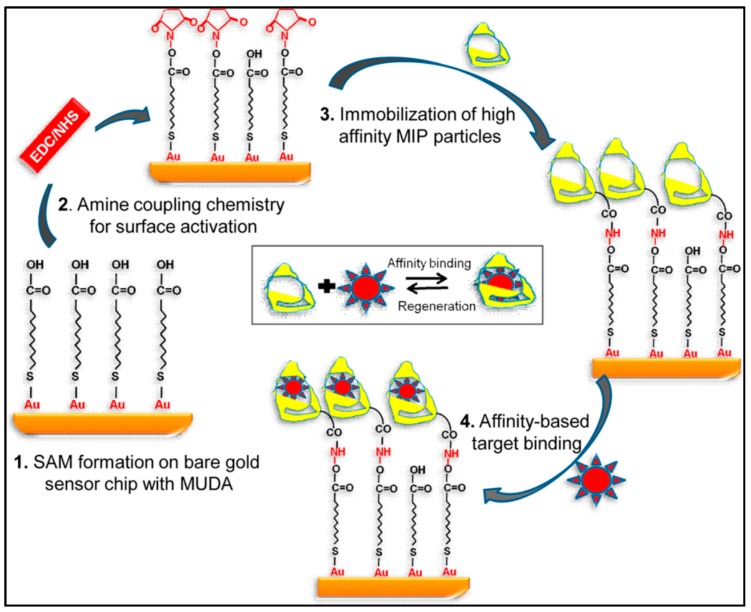
The SPR sensor for virus detection [126].

They initially coated the sensor with mercaptoundeconoic acid to acquire a self-assembled monolayer on the SPR surface. After that, they initiated the surface by using the mixture of N-(3-dimethylaminopropyl)-N′-ethyl carbodiimide and N-hydroxysuccinimide prior to the immobilization of imprinted nanoparticles (Figure 23). Their results showed that this method was really suitable to purify of water sources from biologically harmful factors such as viruses, bacteria, and fungi [126].

Jenik et al. adapted a sensor for detection of human rhinovirus and the foot-and-mouth disease virus [129]. Their procedures were guided by polyurethane layers that depict the geometrical properties of the template. Therefore, the imprinting process guided to an artificial antibody toward viruses, which did not only identify their receptor binding sites, but also discovered the whole virus as an entity.

Wangchareansak and collaborators applied molecular imprinting as a screening tool for diverse influenza subtypes [130]. MIPs for each of the subtypes led to a QCM sensor giving a LOD value as low as 10^5^ particles/mL. Their analysis showed that the responses of the QCM sensor were correlated with the differences in hemagglutinin and neuraminidase patterns from databases.

The self-assembled monolayers were used to sense elements that were virions of poliovirus in a particular manner by Wang et al. [131]. They showed that these sensing elements are comprised of a gold-coated silicon chip with co-adsorbed hydroxyl-terminated alkanethiol molecules and template, where the thiol groups were bound to the substrate and self-assembled into extremely ordered monolayers. The molecules were extracted and the specific cavities were created in the monolayer matrix. All studies about virus detection were summarized in Table 8 according to the different parameters.

QCM and SPR sensors were developed to detect hemagglutinin which is a major protein of influenza A virus by Diltemiz et al. [132]. The authors modified QCM and SPR sensor surfaces with thiol groups and then a monomer mixture was immobilized onto sensor surfaces. They employed aqueous hemagglutinin solutions to determine the detection performance of the sensor for influenza A virus. They also calculated LOD values for QCM and SPR sensors as 4.7 × 10^−2^ μM, and 1.28 × 10^−1^ μM, in the 95% confidence interval. 

## 4. Conclusions

Molecular imprinting can be described as a method for creating synthetic polymers with bio-mimetic molecular recognition capability for diverse templates, where the template directs the positioning and orientation of the structural components via a cross-linking agent, followed by the elution of template to leave cavities which are complementary to the template molecules in shape, size and steric configuration. Macromolecules refer to molecules with molecular weights reaching tens of thousands of Dalton or more. Most macromolecules are made up of simple molecules. For example, the protein units are amino acids, and the nucleotide is the basic unit of nucleic acids. Detection of macromolecules has also been a great concern with the rapid development of biomedicine and proteomics. To establish a fast, simple, specific and high throughput detection approach for macromolecules has become one of the research focuses in analytical science. Compared to the small molecule imprinting, the macromolecular imprinting technique presents more difficulties and challenges. First of all, the elution and recognition of template molecules are the primary issues in the macromolecular imprinting technology because of low elution efficiency of the templates due to their large molecular size. Meanwhile, the highly cross-linked network structure usually limits the diffusion of the target molecule, leading to low binding capacity and long equilibration times. The sensors and recognition elements used in the sensor systems have an enormous impact on various biotechnology applications such as medicine, health-care, the pharmaceutical industry, environmental protection and monitoring, food analysis, defence and security. The combination of electronics and synthetic recognition elements prepared by molecularly imprinted polymers inspired by the natural mechanisms of antigen/antibody interactions has fuelled studies that encompass the development of novel, rapid, reliable, cheap, selective and sensitive diagnostic tools. In particular, the detection of macromolecules has required the development of several sensors that are based on molecularly imprinted polymers with sufficient sensitivity, selectivity and stability. The molecularly imprinted sensors have a “molecular key and lock” principle thus give the researchers the possibility to achieve fast, precise detection of target molecules. A huge mass of knowledge on molecularly imprinted sensors is now available addressing the practical needs for the detection of the broad spectrum of analytes, ranging from small molecules to large molecules like proteins, enzymes, nucleic acids and even whole cells, bacteria and viruses. 

Hue et al. have addressed the perspectives of macromolecular imprinting sensors and their applications, including optical, electrochemical and mass-sensitive molecular imprinting sensors. In addition, the opportunities, challenges, and further research orientations of molecular imprinting sensors for macromolecules detection prospected. Difficulties and challenges arise from the drawbacks of unsatisfactory recognition efficiency due to relatively high mass transfer limitations, and the effective smaller size of the MIP was described by them. They presented a review giving a “visual” overview of the approaches to solving problems related to challenges of the bulk imprinting method and the emergence of surface imprinting and epitope imprinting techniques of the macromolecular imprinting methods to reduce the limitations of imprinting processes [133]. In addition, Iskierkoa and coworkers have focused on gathering, summarizing, and critically evaluating the results of the last decade on the separation and sensing of macromolecular compounds and microorganisms with the use of molecularly imprinted polymer synthetic receptors. Their article mainly considers chemical sensing of deoxyribonucleic acids, proteins and protein fragments as well as sugars and oligosaccharides. Moreover, it briefly discusses the fabrication of sensors for determination of bacteria and viruses that can ultimately be considered as extremely large macromolecules [134]. 

Molecularly imprinted polymers and their incorporation with various transducer platforms are among the most promising approaches for the detection of several macromolecules. The variety of molecular imprinting techniques used for the preparation of biomimetic sensors, including surface imprinting, micro-contact imprinting and epitope imprinting with various transducer platforms such as optical, electrochemical, impedance, current, potential and mass devices were overviewed for different macromolecule types, including enzymes, antibody/antigens, proteins, nucleic acids, cells, bacteria and viruses in detail in this review. 

There is still a lack of good information on the molecular properties of molecularly imprinted polymers and also on means to improve the stability features of structures that are regarded as already very stable. A next step would be the further optimization of active surface and sensing platforms with uniform and easily reachable binding sites to decrease the difficulties in the accessibility of the binding site shape in the three dimensional polymer networks with increased mass transport resulting in adequate recognition properties and also better selectivity and sensitivity for a broader range of analytically significant molecules. The molecularly imprinted sensors are expected to create a revolution in researchers’ understanding of usual sensor devices by providing a new generation of sensing materials.

## Figures and Tables

**Table 1:** Advantages and disadvantages of some recognition molecules in sensors.

Recognition Molecule	Sensor Identification	Advantages	Disadvantages
Enzyme	Enzymatic sensor	- Specificity - Basic equipment and procedures	- High cost and time-wasting purification - Low stability - Efficiency only at an optimum pH and temperature
Antibody	Immunosensor	- Extreme affinity - Specificity	- Minimal target - Hard production - Use of animals requirement - Stability deficiency
Nucleic acid	Genosensor	- Stability	- Minimal target
Cell	Cell sensor	- Low cost preparation - Low purification needs	
Aptamer	Aptasensor	- Easy to modify - Possibility of structure design - Possibility of denaturalization and rehybridization - Possibility of distinguishing targets - Thermal stability - *In-vitro* synthesis	
MIPs	MIP sensor	- Extreme thermal, chemical and mechanical resistance Reusability	- Complicated fabrication methods - Time-wasting procedure - Incompatibility with aqueous solutions - Template release - Lower specificity

**Table 2:** Comparison of the sensor studies for enzyme detection.

Parameters	Reference		
	[89]	[90]	[91]
Target	Lysozyme	Lysozyme	Lysozyme
Linear dynamic range	21–1400 nM	1–500 nM	0–3 µM
Buffers (Ads, Des)	pH 7.4, 1 M NaCl (pH 8.0)	pH 7.0, ethylene gycol	10 mM Tris/HCl (pH 7.4), NA
Time	45 min	23 min	NA
Limit of detection	32.2 nM	0.66 nM	NA

NA: not available.

**Table 3:** Comparison of the sensor studies for antibody and antigen detection.

Parameters	Reference						
	[65]	[92]	[93]	[94]	[95]	[96]	[97]
Target	Fab fragment	Cyclic citrullinated peptide antibody	Hepatitis B surface antibody	Prostate specific antigen	Prostate specific antigen	Vancomycin	Immunoglobulin G
Linear dynamic range	2–15 mg/mL	1–200 RU/mL	0–120 mIU/mL	0.1–50 ng/mL	0.29–5000 ng/mL	0.001–70 nM	0.05–2.0 mg/mL
Buffers (Ads, Des)	pH 7.4, 1 M NaCl	pH 7.0, Acetic acid with Tween 20	NA, 1 M Ethylene glycol	pH 7.4, Glycine HCl (pH 2.5)	PBS, 100 mM HCl	PBS (pH 7.4), NA	pH 7.4, 1 M NaCl
Time	40 min	40 min	40 min	50 min	53 min	NA	52 min
Limit of detection	56 ng/mL	0.177 RU/mL	208.2 mIU/mL	91 pg/mL	0.29 ng/mL	2.5 pM	NA

NA: not available.

**Table 4:** Comparison of the sensor studies for protein detection.

Parameters	Reference									
	[60]	[99]	[100]	[101]	[102]	[103]	[104]	[105]	[106]	[107]
Target	Myoglobin	Myoglobin	Lipoprotein	Bovine hemoglobin and trypsin	Protein C	Bovine hemoglobin	Hemoglobin and myoglobin	Myoglobin	Bilirubin	Cytochrome C
Linear dynamic range	0.1–10 µg/mL	0.5–53.3 µg/mL	4–400 mg/dL	3 mg/mL	0.1–30 µg/mL	1 × 10^−3^–1 × 10^−10^ g/L	0–250 µg/mL	1.24 × 10^−6^–3.71 × 10^−7^ mol/L	1.71–85.51 µM	1–500 µg/mL
Buffers (Ads, Des)	pH 7.4, 1 M Ethylene glycol	pH 5.0, Proteinase K (pH 7.4)	pH 7.4, Acetic acid (10%) nd SDS (0.1%)	NA	pH 5.0, 0.5 M NaCl (pH 5.0)	pH 7.0, NA	Dulbecco’s PBS (pH 7.15), NA	HEPES (pH 4.0), oxalic acid	pH 11.0, 2 M NaOH, 1 M Na_2_CO_3_, 25 mM EDTA	pH 7.4, Tween-20 (0.1%)
Time	27 min	NA	60 min	50 min	30 min	120 min	2–10 min	<15 s	25 min	7.5 min
Limit of detection	87.6 ng/mL	0.827 µg/mL	4 mg/mL	NA	0.01 µg/mL	3.09 × 10^−11^ g/L	NA	1.3 × 10^−6^ mol/L	0.45 µg/mL	NA

NA: not available.

**Table 5:** Comparison of the sensor studies for nucleic acid detection.

Parameters	Reference				
	[109]	[110]	[111]	[112]	[113]
Target	Guanosine and guanine	8-Hydroxy-2-deoxyguanosine	Thymine	Myoglobin	Myoglobin
Linear dynamic range	20–400 µmol/L	0.1–1 mM	10–100 µM	0–80 nM	0.0001–0.2 g/L
Buffers (Ads, Des)	PBS, 0.1 M glycine–HCl (pH 2.2)	pH 10.0, 0.1 M Glycine–HCl	pH 7.4 HBS, 0.5 M NaCl	pH 7.4, NA	PBS, NA
Time	NA	30 min	60 min	45 min	120 min
Limit of detection	NA	0.0125 µM		27 pM	26.3 ng/mL

NA: not available.

**Table 6:** Comparison of the sensor studies for cell detection.

Parameters	Reference			
	[115]	[116]	[117]	[118]
Target	*M. tuberculosis* bacilli	Chinese hamster ovarian cell	Macrophage and cancer cell	Mesenchymal stem cells
Linear dynamic range	NA	NA	10^4^–10^6^ cells/mL	30 × 10^3^ cells
Buffers (Ads, Des)	pH 7.4, NA	pH 7.4, NA	pH 7.4, SDS (0.1%)	NA
Time	10 min	45 min	67 min	NA
Limit of detection	NA	NA	10^4^ cells/mL	NA

NA: not available.

**Table 7:** Comparison of the sensor studies for bacteria detection.

	Reference	Reference	Reference	Reference	Reference	Reference
Parameters	[120]	[121]	[122]	[123]	[124]	[125]
Target	*E. coli*	*E. coli*	Endotoxin from *E. coli*	*S. cerevisiae*	Bacteria	Marine pathogen sulfate-reducing bacteria
Linear dynamic range	1 × 10^2^–1 × 10^7^ CFU/mL	0.5–4.0 McFarland	4.4–5.3 × 10^−10^ M	2 × 10^7^–5 × 10^9^ cells/mL	10^7^ cfu/mL	1.0 × 10^4^–1.0 × 10^8^ cfu/mL
Buffers (Ads, Des)	pH 7.4, NA	pH 7.4, Ethyl alcohol, 1 M lysozyme	HEPES (pH 7.0), NA	pH 6.0,	PBS, NA	0.2 M PBS buffer (pH 7.4), NA
Time	NA	7 min, 20 min	3.5 min	60 min	NA	6 min
Limit of detection	70 CFU/mL	3.72 × 10^5^ CFU/mL, 1.54 × 10^6^ CFU/mL	0.44 ng/mL	1.0 × 10^4^ cells/mL	NA	0.7 × 10^4^ cfu/mL

NA: not available.

**Table 8:** Comparison of the sensor studies for virus detection.

Parameters	Reference				
	[126]	[129]	[130]	[131]	[132]
Target	Waterborne virus	Picornavirus	Influenza A virus	Poliovirus	Influenza
Linear dynamic range	0.33–27 pmol	100–300 µg/mL	2.5 × 10^7^–5 × 10^5^ particles/mL	0–5000 × 10^8^ particles/mL	0.01–0.16 mM
Buffers (Ads, Des)	PBS, 0.1 M HCI and 20 mM NaOH	10 mM Tris (pH 7.2), NA	pH 7.2, NA	Dulbecco’s PBS (pH 7.15), NA	HEPES (pH 7.4), Glycine/HCl (pH 2.0)
Time	8 min	75 min	80 min	60 min	25 min, 30 min
Limit of detection	5 × 10^6^ pfu/mL	NA	10^5^ particles/mL	NA	4.7 × 10^−2^ μM, 1.28 × 10^−1^ μM

NA: not available.
